# Supplementary material for: The African swine fever virus gene MGF_360-4L inhibits interferon signaling by recruiting mitochondrial selective autophagy receptor SQSTM1 degrading MDA5 antagonizing innate immune responses
Source: mBio. 2025 Feb 25;16(4):e02677-24. doi: 10.1128/mbio.02677-24 (PMC11980378; doi:10.1128/mbio.02677-24)
Supplement: Supplemental figures — Figures S1-S5. [file mbio.02677-24-s0002.docx]

**
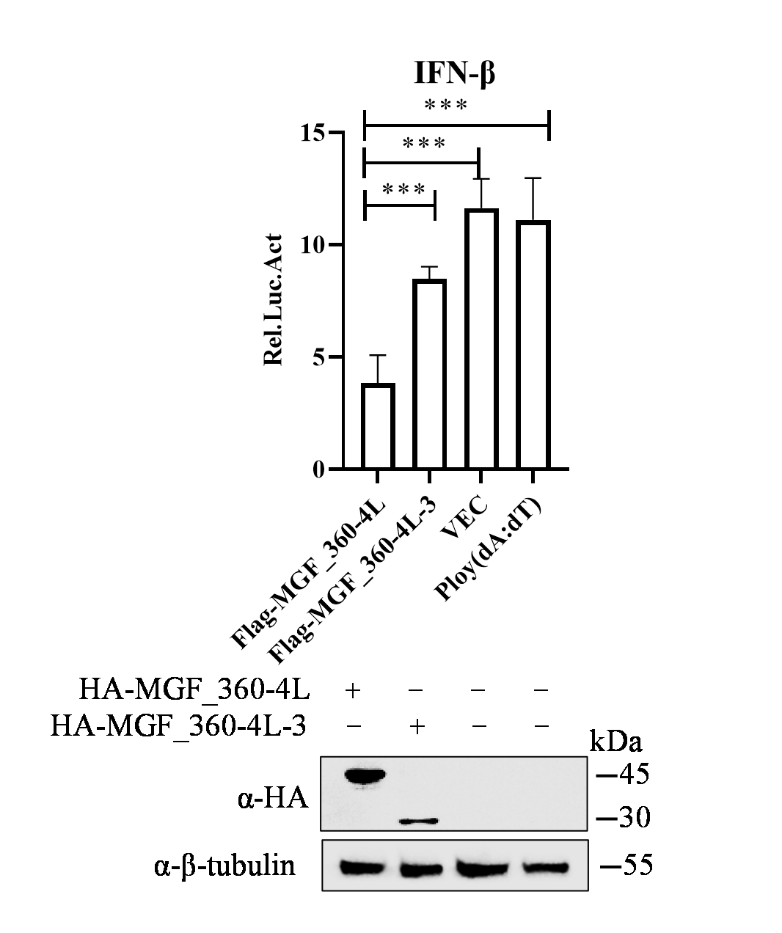
**

Figure S1. The deletion of the alpha-lactotoxin LT1a-like domain in MGF_360-4L attenuates its inhibitory effect on IFN-β promoter activation. Transfect HEK293T cells with either pHA-MGF_360-4L or pHA-MGF_360-4L-3, or an empty vector, simultaneously with the TK and IFN-β luciferase reporter plasmids. All cells are treated with Poly(dA:dT) stimulation, and luciferase activity is measured to indicate IFN-β activation.


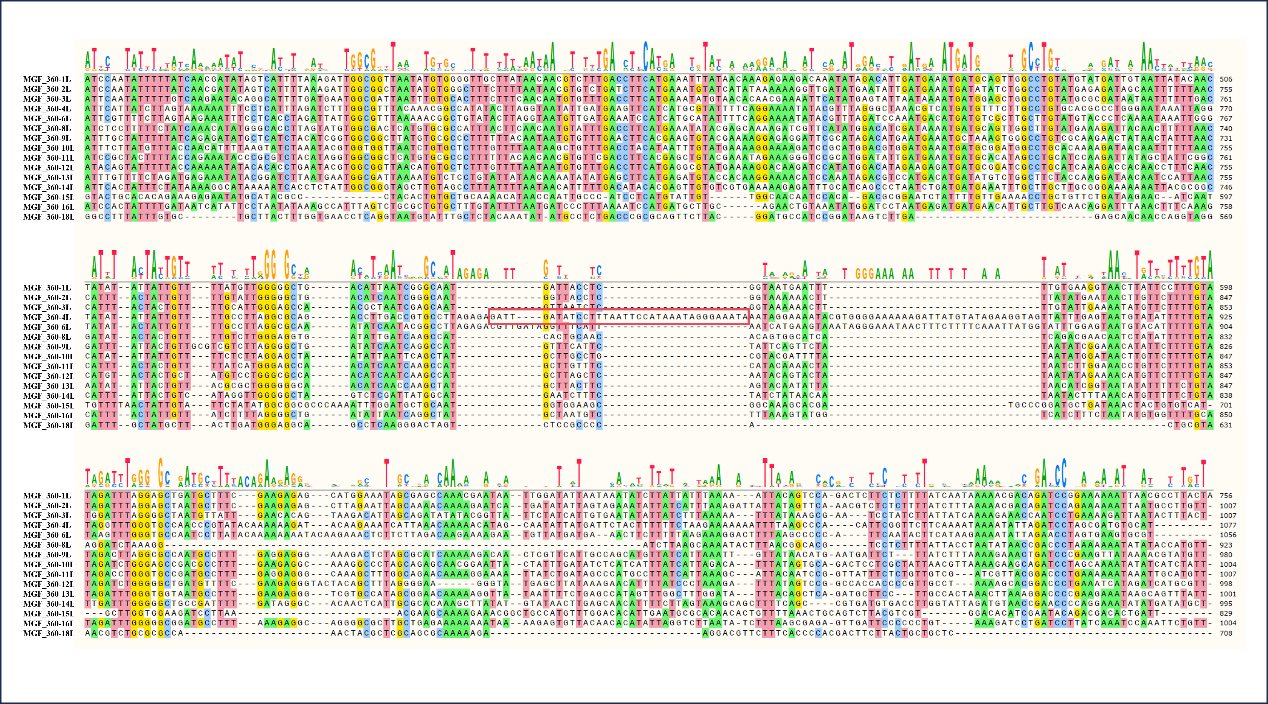


Figure S2. MGF_360 family gene sequence alignment with Alpha-lactotoxin_LT1a domain. Comparison of the coding region nucleotide sequences of all 15 members in the MGF_360 family (GenBank accession number: MK333180.1) reveals the differences in their base sequences. The sequence comparison region highlights the coding area for the Alpha-lactotoxin LT1a-like domain in MGF_360-4L. The red box indicates the unique nucleotide sequence insertions specific to MGF_360-4L.

**
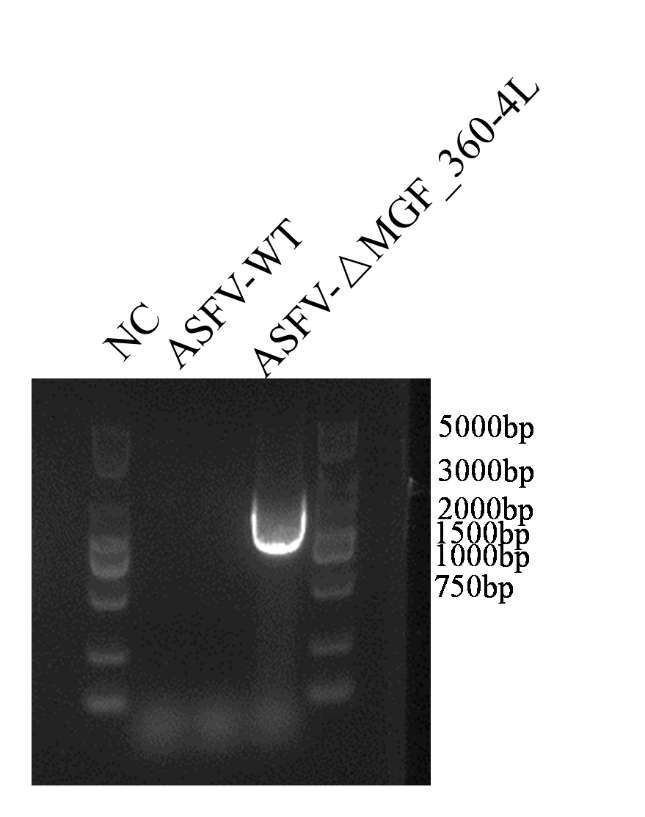
**

Figure S3. Identification and purity assessment of ASFV-△MGF_360-4L.


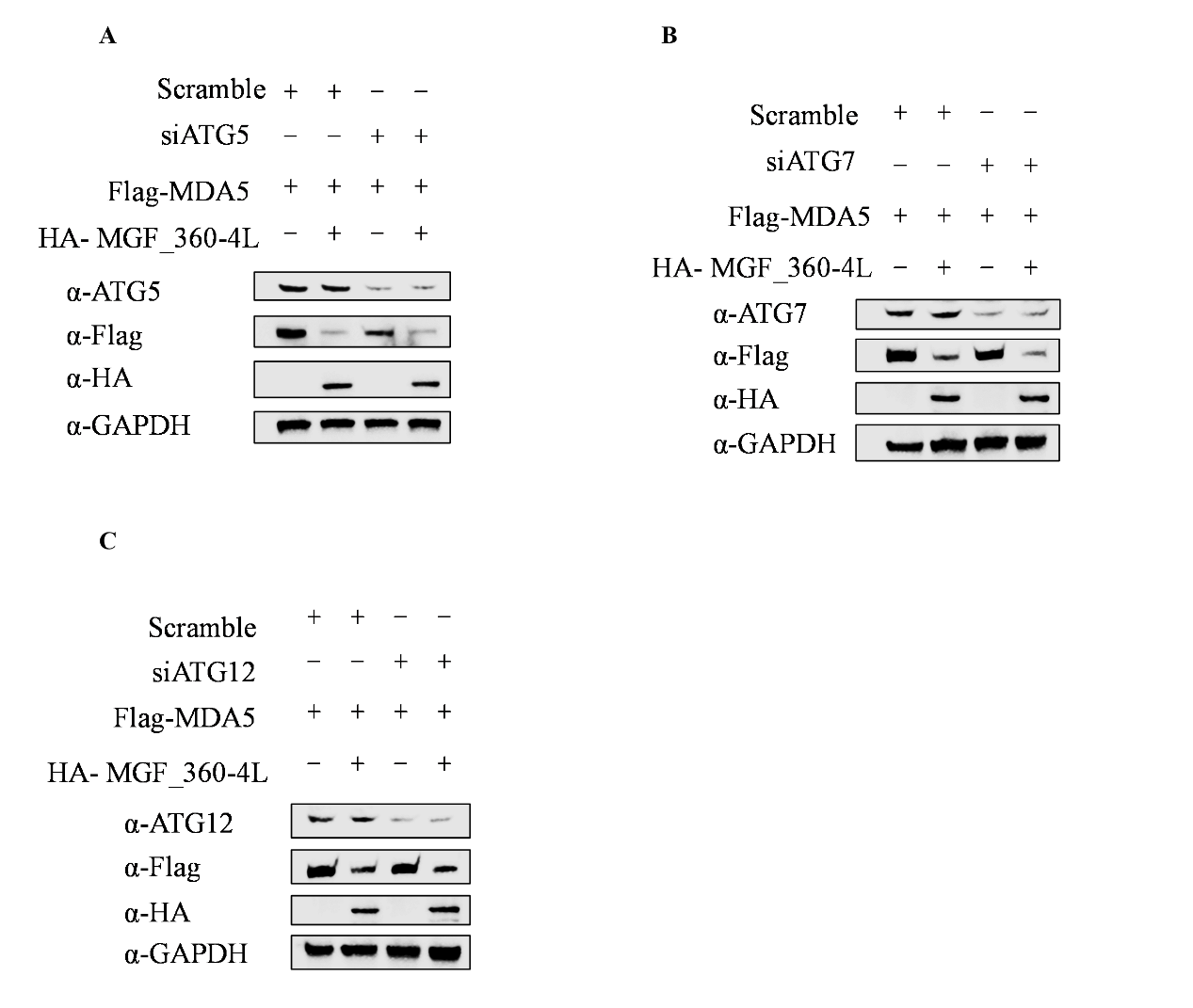


Figure S4. ATG5, ATG7, and ATG12 do not affect the autophagic degradation of MGF_360-4L. (A-C) HEK293T cells were transfected with pFlag-MDA5 or pHA-MGF_360-4L along with an empty vector and simultaneously transfected with siATG5 (A), siATG7 (B), siATG12 (C) or scramble. After 24 hours, protein expression was assessed by Western blotting.

**
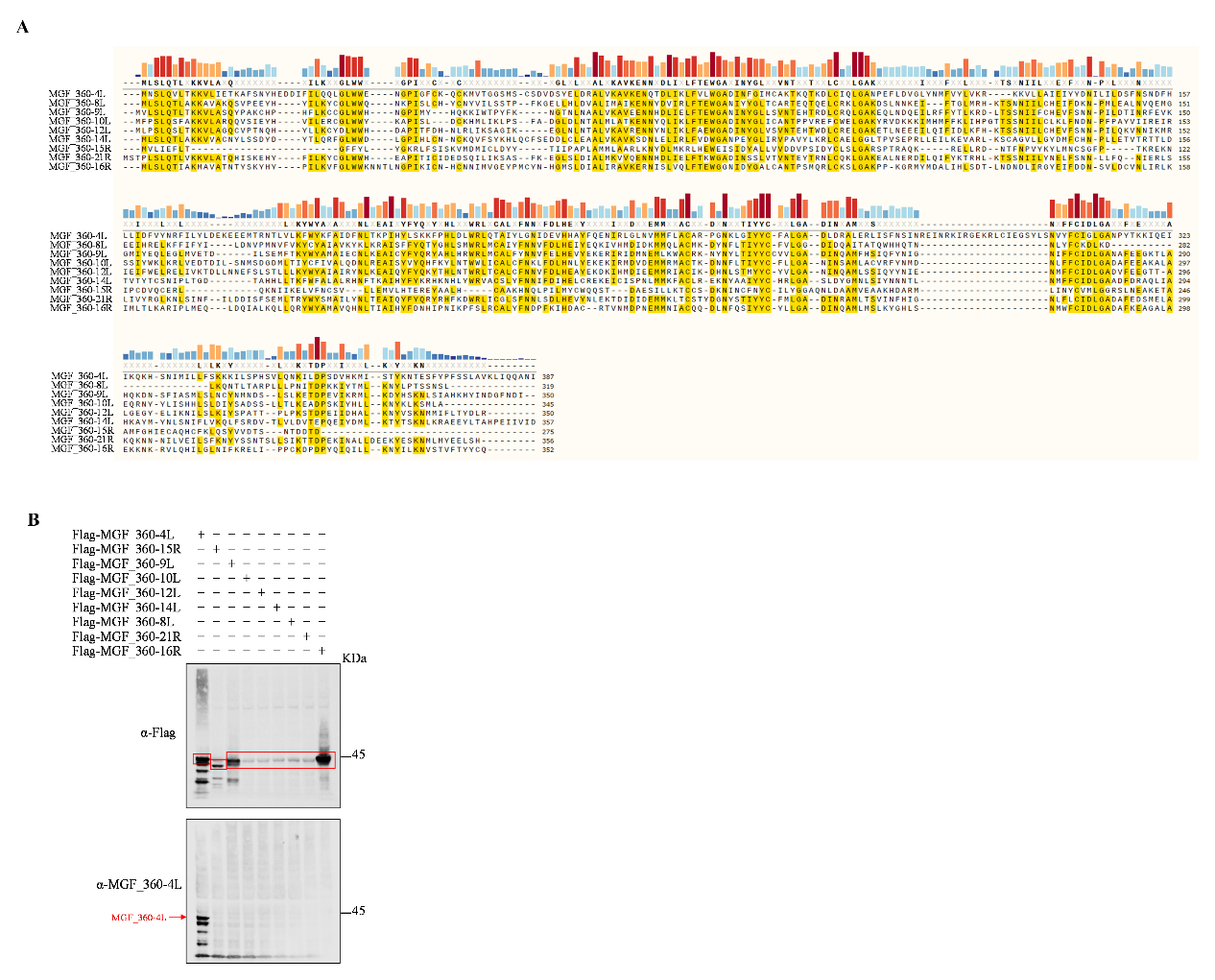
**

Figure S5. Amino acid sequence characteristics of selected MGF_360 family members and verification of MGF_360-4L antibody specificity. (A) To compare the amino acid sequences of several MGF_360 family members, sequences of MGF_360-4L, MGF_360-15R, MGF_360-9L, MGF_360-10L, MGF_360-12L, MGF_360-14L, MGF_360-8L, MGF_360-21R, and MGF_360-16R were retrieved and aligned using MUSCLE 3.8.1551 software. Highly conserved regions across the sequences are highlighted in orange. The degree of amino acid similarity in the aligned regions is depicted using bar graphs, with blue indicating low similarity and dark red indicating high similarity. (B) To assess the specificity of the MGF_360-4L antibody, HEK-293T cells were transfected with plasmids encoding Flag-tagged MGF_360 family proteins: MGF_360-4L, MGF_360-15R, MGF_360-9L, MGF_360-10L, MGF_360-12L, MGF_360-14L, MGF_360-8L, MGF_360-21R, and MGF_360-16R. After 24 hours post-transfection, cells were incubated with anti-Flag or MGF_360-4L antibodies. The specificity of the MGF_360-4L antibody was verified by observing its reactivity with MGF_360-4L but not with the other MGF_360 family members.
