# Supplementary material for: The African swine fever virus gene MGF_360-4L inhibits interferon signaling by recruiting mitochondrial selective autophagy receptor SQSTM1 degrading MDA5 antagonizing innate immune responses
Source: mBio. 2025 Feb 25;16(4):e02677-24. doi: 10.1128/mbio.02677-24 (PMC11980378; doi:10.1128/mbio.02677-24)
Supplement: Supplemental table — Table S1. [file mbio.02677-24-s0003.docx]

| Table S1 The primers used in this study | | | | |
| --- | --- | --- | --- | --- |
| Target | | Sequences of primers (5’-3’) | | References or GenBank Accession Numbers |
| Procine-ISG15 | F: aacgacaagggtcgcagcaa | | NM_001128469.3 | |
|  | R: tccatgggcttcccctcgaa | |  |  |
| Porcine-OAS1 | F: cgaccctcaggggccgatca | | NM_214303.2 | |
|  | R: cagggcatcaaaggcgggca | |  |  |
| Porcine-OASL | F: tcttccgtgccatcgccttc | | NM_001031790.1 | |
|  | R: gagggggcagcctagcaatg | |  |  |
| Porcine-MX1 | F: cgtcgatccggctccacttc | | AB259856.1 | |
|  | R: gatgtggctggagatgcgct | |  |  |
| Porcine-IFIT2 | F: gacccggctcaagtgtggag | | NM_001315658.1 | |
|  | R: attccgaggaggtggccagt | |  |  |
| Porcine-IFIT1 | F: cctggggcaactatgcctgg | | HQ679904.1 | |
|  | R: aagcagaccttggcccgttc | |  |  |
| Porcine-STAT1 | F: aatgccggcgccagaaccaa | | NM_213769.1 | |
|  | R: agggccagcagtgggaccaa | |  |  |
| Porcine-STAT2 | F: gcgccggctgctgaagaaga | | AB004061.1 | |
|  | R: agcctcgtcccgggggattc | |  |  |
| Porcine-GAPDH | F: gcgagatcccgccaacatca | | NM_001206359.1 | |
|  | R: gtccctccacgatgccgaag | |  |  |
| Porcine-RIG-I | F: atgacagcagagcagcggcg | | NM_213804.2 | |
|  | R: gggaatgggcaaccttgagtga | |  |  |
| Porcine-MDA5 | F: atgtcgtcggatgggtattc | | MF358967.1 | |
|  | R: gatgaggaccccatcaccagctag | |  |  |
| Porcine-MAVS/VISA | F: atgacgtttgccgaggacaagac | | MK302496.1 | |
|  | R: gtaccggcggcgcctgccccagtga | |  |  |
| Porcine-TRAF3 | F: atgacacacagaatggagccgggtca | | XM_021081623.1 | |
|  | R: ggatacttcggatctgcctgacccctga | |  |  |
| Porcine- IKKε | F: atgagctggtcaccttccctgac | | XM_054360427.1 | |
|  | R: acctccgcttcccgggttcaagtga | |  |  |
| Porcine-cGAS | F: atggcggcccggcggggaaagtc | | XM_013985148.2 | |
|  | R: caatggatttccagttttttggtga | |  |  |
| Porcine-STING/VISA | F: atgccctactccagcctgcatccatc | | FJ455509.1 | |
|  | R: cttccactccgctcagatatcttctga | |  |  |
| Porcine-IRF3 | F: atgggaactcagaagcctcggatcct | | NM_213770.1 | |
|  | R: tggtggaggacatggatttctag | |  |  |
| Porcine-IRF7 | F: atggccgcggctcctgacagggggtgc | | NM_001097428.1 | |
|  | R: gatggaggtggagcagccggcctag | |  |  |
| Porcine-ISG15 | F: atgggtagggaactgaaggtgaag | | NM_001128469.3 | |
|  | R: gggagcacctcaccgagtgctag | |  |  |
| Homo-OPTN | F: atgtcccatcaacctctcagctgcc | | KR710298.1 | |
|  | R: agattcacgtgatggattgcatcatt | |  |  |
| Homo-NBR1 | F: at ggaaccacaggttactctaaatgtgact | | NM_005899.5 | |
|  | R: acaacgactggtacagccaacgctattga | |  |  |
| Homo-TOLLIP | F: atggcgaccaccgtcagcactca | | AY730683.1 | |
|  | R: gctgcagatgggggaggagccatag | |  |  |
| Homo-NDP52 | F: atggaggagaccatcaaagatccccc | | U22897.1 | |
|  | R: tgttctgccactctctctga | |  |  |
| Homo-SQSTM1/p62 | F: atggccatgtcctacgtgaaggatg | | KR709835.1 | |
|  | R: gt attcaaagcatcccccgccgttg | |  |  |
| ASFV-MGF_360-4L | F: ATGAACTCTTTACAGGTTCT | | MK333180.1 | |
|  | R: TTAGATGTTTGCTTGTTGTA | |  |  |
| MGF_360-4L-L | F: tcatataatgaatatatggt | | MK333180.1 | |
|  | R: ATATATAATGTTATAAAAATA | |  |  |
| MGF_360-4L-R | F: cgtattattatgaataacagg | | MK333180.1 | |
|  | R: caatacttcataagaaaata | |  |  |
| mCheery | F: ATGGTGAGCAAGGGCGAGGAG | | MN007111.1 | |
|  | R: CTTGTACAGCTCGTCCATGCC | |  |  |
